# Absence of Evidence of Rift Valley Fever Infection in *Eulemur fulvus* (Brown Lemur) in Mayotte During an Interepidemic Period

**DOI:** 10.1089/vbz.2016.2079

**Published:** 2017-05-01

**Authors:** Raphaëlle Métras, Laure Dommergues, Katia Ortiz, Marion Pannequin, Christian Schuler, Patrick Roux, John W. Edmunds, Matt J. Keeling, Catherine Cêtre-Sossah, Eric Cardinale

**Affiliations:** ^1^ Department of Infectious Disease Epidemiology, London School of Hygiene and Tropical Medicine, London, United Kingdom.; ^2^ GDS Mayotte-Coopérative Agricole des Eleveurs Mahorais, Coconi, Mayotte, France.; ^3^ Museum National d'Histoire Naturelle UMR 7205, Institut de Systématique, Evolution, Biodiversité, ISYEB, Réserve Zoologique de la Haute-Touche, Obterre, France.; ^4^ Clinique Vétérinaire de Mamoudzou, Mamoudzou, Mayotte, France.; ^5^ Warwick Infectious Disease Epidemiology Research, Warwick University, Coventry, United Kingdom.; ^6^ UMR CMAEE, CIRAD, Sainte-Clotilde, La Réunion, France.; ^7^ UMR1309 CMAEE, INRA, Montpellier, France.

**Keywords:** arbovirus, emerging diseases, epidemiology, lemurs, Rift Valley fever, wildlife

## Abstract

The potential role of *Eulemur fulvus* (brown lemur) in the epidemiology of Rift Valley fever (RVF) in Mayotte, during an interepidemic period, was explored. In February and March 2016, 72 animals were blood sampled and tested for RVF. No evidence of RVF genome or antibodies was found in the samples. The role of other wild mammals on the island should, however, be further investigated.

## Introduction

**FIG. 1.**
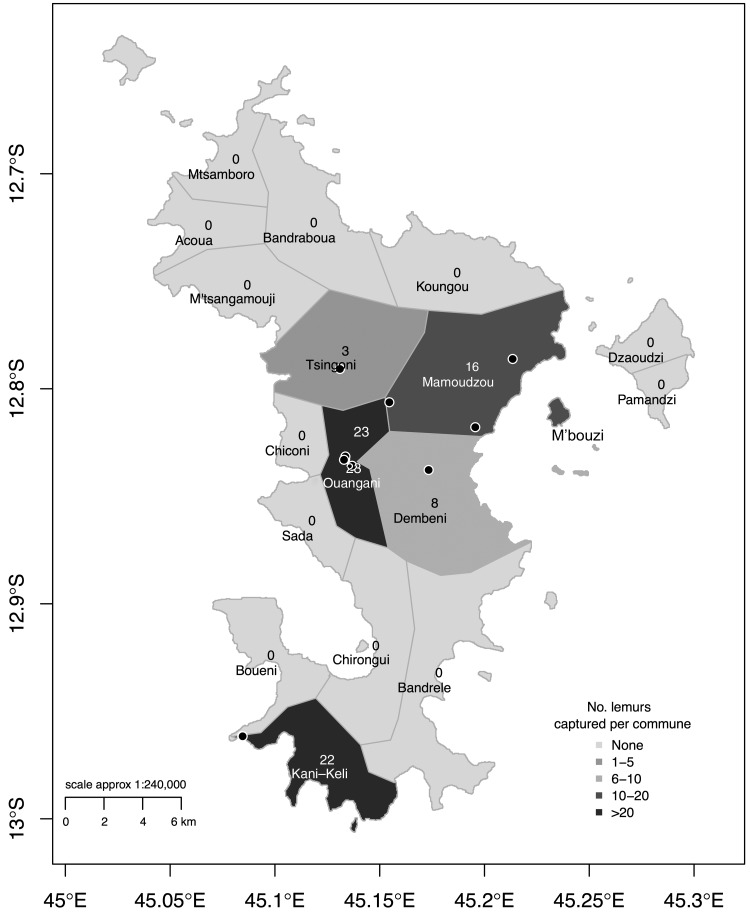
The islands of Mayotte (374 km^2^). Number of lemurs sampled per commune and location of capture sites in the 2016 study (*dots*). The 2012 study (Lernout et al. 2013) was held on the M'bouzi islet, free of living domestic mammals and humans.

A re-emergence of Rift Valley fever (RVF) in livestock was observed on the island of Mayotte in 2008–2010. Since then, the virus may have circulated at a low level, indicating a current interepidemic period (Métras et al. 2016). The mechanisms and reservoirs for RVF virus persistence between epidemics still remain unclear. Although vertical transmission in *Aedes* spp. mosquitoes has been demonstrated (Linthicum et al. 1985), it is possible that wild mammals could play a role in virus persistence (Olive et al. 2012). Understanding the role of wildlife, therefore, remains an important question (Bird and McElroy 2016). Mayotte is small (374 km^2^, Fig. 1), with only a few wild mammalian species described, such as bats (*Pteropus seychellensis comorensis*, *Chaerephon pusillus*, and *C. leucogaster*), rodents (*Rattus rattus* and *Mus musculus*), tenrecs (*Tenrec ecaudatus*), civets (*Viverricula indica*), shrews (*Suncus madagascariensis*), and lemurs (*Eulemur fulvus*) (brown lemur, Order *Primates*, Family *Lemuridae*). Previous studies in wildlife indicated that bats and micromammals could be involved in RVF epidemiology (Olive et al. 2012). Serological evidence of past arboviral infections in lemurs, such as Chikungunya or Flaviviruses (Fontenille et al. 1988, Vourc'h et al. 2014), has been found from Madagascar and Mayotte, suggesting that mosquitoes and especially *Aedes* spp. can bite these animals and infect them with other pathogens, such as RVF virus. In Mayotte, in 2012, 50 lemurs, living on the M'bouzi islet (separate from Mayotte main island, Fig. 1), were found serologically negative for RVF antibodies (Lernout et al. 2013). As this islet is not inhabited by humans nor domestic animals, exposure to RVF during the 2008–2010 epidemic was not expected. In this study, we investigated whether the population of brown lemurs from the main island of Mayotte had been exposed to RVF during the past epidemic, and whether they could play a role in virus persistence during an interepidemic period.

## Methods

The study was reviewed and approved by DEAL (Direction de l'Environnement Aménagement et Logement) of Mayotte (AP268/DEAL/SEPR/2015, delivered on October 22, 2015), the Ethics Committee of the CYROI La Reunion (APAFIS-2015110512417776, delivered on November 9, 2015), and by the Animal Welfare Ethical and Review Board of the London School of Hygiene and Tropical Medicine (2015/3N).

A cross-sectional study was conducted during February and March 2016. A total of 72 brown lemurs, collected over 12 days, from nine different capture sites, in five communes (Fig. 1), were captured, blood sampled, and released. The whole island had been previously affected by RVF (Cetre-Sossah et al. 2012). The selection of the capture sites attempted to cover the main island, taking into account site accessibility, known regular presence of lemurs, and logistical constraints. Brown lemurs in Mayotte live in trees and in groups of varying size of 5–15 individuals. Animals were caught in groups, using large fruit-baited cages (H160 × W120 × L200 cm). Once in the cage, the animals were caught one by one using a net and anesthetized with a routine dose of 0.125 mg/kg of medetomidine and 7.5 mg/kg ketamine by intramuscular injection in the thigh. During the anaesthesia, 3 mL of blood was withdrawn from the femoral vein. Data on age (<1 year old, 1–3 years old, 3–6 years old, and >6 years old) were collected on 70 animals. The estimation of age was based on the examination of the teeth and the development of the primary and secondary sexual characteristics. Animals were kept under surveillance until full recovery and were released on the same day, at the site of capture. Blood samples were centrifuged and sera were kept at −80°C and shipped to La Reunion (France) for laboratory testing. To look for acute infections, detection of RVF virus genome was performed using the RT-PCR technique based on the L segment with a detection limit of 0.5 TCID_50_/mL (Wilson et al. 2013). Evidence of past infections was examined by detecting RVF-specific IgG antibodies in serum (ID Screen RVF Competition multispecies ELISA kit; IDVet, Grabels, France) with a diagnostic sensitivity of 98% and specificity of 100% (Kortekaas et al. 2013).

## Results

Fourteen percent (*n* = 10) of animals were estimated to be <1 year old, 23% (*n* = 16) were between 1 and 3 years old, 36% (*n* = 25) were aged 3–6 years, and 27% (*n* = 19) were estimated to be >6 years old. All samples were found negative by RT-PCR and ELISA, in all age groups, indicating no evidence of current, recent, or past RVF infections in our study animals [seroprevalence *p* = 0, 95% confidence interval (0–0.05)]. The estimated lemur population size of Mayotte is about 10–20,000 (Tarnaud 2012), and our study sample, although small, would have allowed to detect an estimated seroprevalence of 15%, with a 10% precision at a 95% confidence level (with a design effect of 1.5).

## Discussion

There is evidence that lemurs can be exposed to Chikungunya and Flavivirus infections in Mayotte and Madagascar (Fontenille et al. 1988, Vourc'h et al. 2014). All our study animals were found negative to RVF. Although the lemur population on the island might have been infected by RVF during the epidemic phase, it seems very unlikely that they play a major role in the interepidemic persistence of RVF virus in Mayotte. In addition, despite the ELISA test used had not been validated with lemurs, it is not species specific, and therefore gives weight to the results obtained. Finally, as previous surveys of lemurs in Mayotte did not target populations sharing an environment with RVF-seropositive livestock and humans (Lernout et al. 2013), our article presents the first study investigating the potential role of lemurs in the epidemiological cycle of RVF virus in Mayotte. Although tenrecs (Family *Tenrecidae*) and rodents (Family *Nesomyidae* and *Muridae*) in neighboring Madagascar showed no evidence of RVF infection during 2008–2010 after the RVF epidemic (Olive et al. 2013), similar serological studies in other wild mammals would be necessary in Mayotte to assess their role in RVF persistence.
